# 230. A Novel SARS-CoV-2 mRNA Virus-Like Particle Vaccine is Highly Potent and Well Tolerated in Adults in a Phase I Randomized Clinical Trial

**DOI:** 10.1093/ofid/ofaf695.082

**Published:** 2026-01-11

**Authors:** Tope Oyedele, Rachel Park, Kelly Morales, Manish Jain, Lawrence Sher, Apinya Vutikullird, Thomas Klein, Abby Isaacs, Kathryn Shoemaker, Ann Marie Stanley, Joseph Lee, Cindy Handelsman, Stacey Cromer Berman, Lee-Jah Chang

**Affiliations:** AstraZeneca, Gaithersburg, MD; AstraZeneca, Gaithersburg, MD; Coastal Carolina Research Center, North Charleston, South Carolina; Flourish - Great Lakes Clinical Trials, Chicago, Illinois; Peninsula Research Associates, Rolling Hills Estates, California; Ark Clinical Research, Long Beach, California; Alliance for Multispecialty Research, East Witchita, Kansas; AstraZeneca, Gaithersburg, MD; Biometrics, Vaccines & Immune Therapies, BioPharmaceuticals R&D, AstraZeneca, Gaithersburg, MD, USA, Gaithersburg, Maryland; Translational Medicine, Vaccines & Immune Therapies, BioPharmaceuticals R&D, AstraZeneca, Gaithersburg, MD, USA, Gaithersburg, MD; AstraZeneca, Gaithersburg, MD; AstraZeneca, Gaithersburg, MD; AstraZeneca, Gaithersburg, MD; AstraZeneca, Gaithersburg, MD

## Abstract

**Background:**

There is a need for coronavirus disease 2019 (COVID-19) vaccines with improved potency and durability, lower reactogenicity, and broader coverage against emerging SARS-CoV-2 variants. This ongoing, Phase I, open-label, randomized, active-controlled study examined the safety and immunogenicity of two novel SARS-CoV-2 mRNA virus-like particle (VLP) vaccines encoding Omicron BA.4/5 (AZD9838) and Omicron XBB.1.5 (AZD6563).

**Figure 1.**
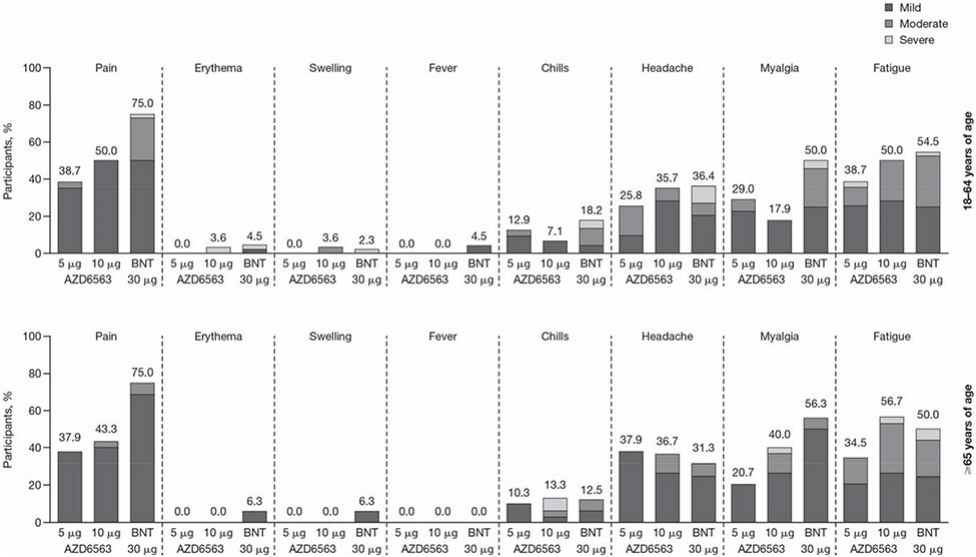
Reactogenicity-associated adverse reactions by severity in AZD6563 or BNT162b2 recipients aged 18–64 and ≥65 years

The number above each bar denotes the percentage of participants reporting the event with any severity. Solicited adverse reactions were collected in a daily eDiary on the day of vaccination (Day 1) through 7 days post-vaccination (Day 8).

BNT, BNT162b2.

**Figure 2.**
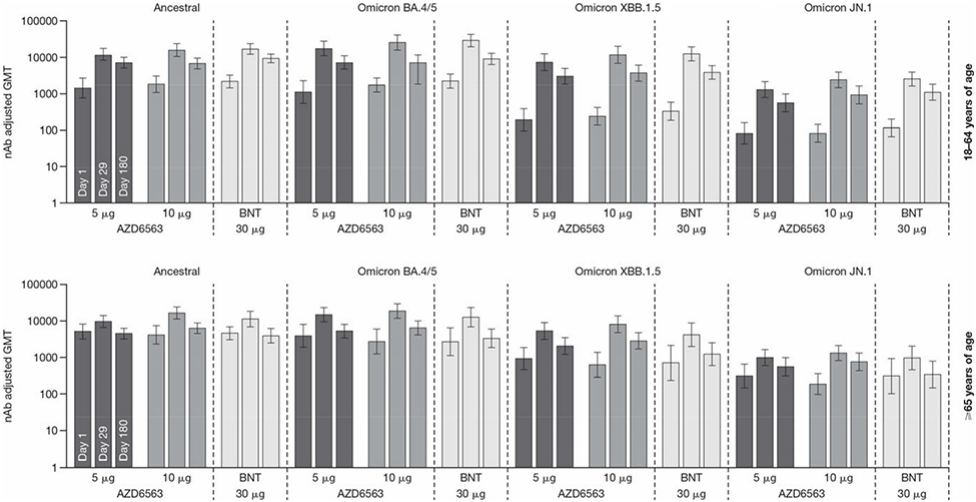
nAb adjusted GMTs against ancestral, Omicron BA.4/5, Omicron XBB.1.5, and Omicron JN.1 in AZD6563 or BNT162b2 recipients aged 18–64 and ≥65 years BNT, BNT162b2; GMT, geometric mean titer; nAb, neutralizing antibody.

**Methods:**

Participants who had natural immunity via previous infection or primary series vaccination with a SARS-CoV-2 vaccine were randomized to receive a single intramuscular injection of 5 or 10 μg AZD6563, 5 or 10 μg AZD9838, or 30 µg BNT162b2, a licensed SARS-CoV-2 mRNA XBB.1.5 variant vaccine.

Solicited adverse reactions (ARs) were collected through Day 8 and unsolicited adverse events (AEs) were collected through Day 29. Serious AEs (SAEs), medically attended AEs (MAAEs), and AEs of special interest (AESIs) were collected through Day 180 at the time of this analysis. Neutralizing antibody (nAb) titers against the ancestral and Omicron variants BA.4/5, XBB.1.5, and JN.1 were measured at baseline, Day 29, and Day 180. All comparisons were descriptive.

**Results:**

Overall, 166 healthy adults aged 18‒64 years and 76 adults aged ≥ 65 years were vaccinated. AZD9838 and AZD6563 were well tolerated at both dosages, with a lower proportion of injection site pain and muscle aches reported among AZD6563 recipients compared with BNT162b2 (Figure 1). Unsolicited AEs were similar between groups. No related AESIs, MAAEs, or SAEs were reported.

Geometric mean titers (GMTs) of the nAbs peaked at Day 29 and remained above baseline at Day 180. Day 29 nAb levels increased with increasing dosages of AZD9838 and AZD6563. In both age groups, 10 μg AZD6563 induced GMTs similar to those observed with 30 μg BNT162b2 at Day 29 and Day 180 (Figure 2).

**Conclusion:**

Two candidate SARS-CoV-2 mRNA VLP vaccines, AZD9838 and AZD6563, were well tolerated, with fewer ARs reported compared with the licensed COVID-19 vaccine BNT162b2. Additionally, 10 μg AZD6563 demonstrated similar immunogenicity to 30 μg BNT162b2, with titers remaining above baseline through Day 180.

**Disclosures:**

Tope Oyedele, MD, AstraZeneca: Employee|AstraZeneca: Stocks/Bonds (Public Company)

